# Selections and Abstracts

**Published:** 1904-02

**Authors:** 


### SELECTIONS AND ABSTRACTS.

### The Care and Treatment of Crippled and Deformed Children.*

One of the most fascinating thoughts in modern surgery is that of the possibility of curing a serious disease by a harmless operation which lasts a comparatively short time, as it were, "by one fell swoop." In this category the dictum of "an inch and a half incision with a week and a half in bed," finds its place. Though this saying may hold true for certain picked cases, mild appendicitis for instance, one may be compelled to make a large incision, create a great deal of disturbance in the relationship of the parts, as when masses of adhesions must be separated, then add to this tamponage and drainage, and the period of convalescence jumps from a week and a half to thrice as many months, and the life of the patient and the success of the operation, together with the thoroughness of the cure, depend as much upon the after-care as upon the operation itself. This is true in the province of orthopedic surgery. Here, then, in this class of surgery the conditions, the tendencies of growth toward deformity, and new growths and deposits often existing from the very beginning of life are met with and demand permanent correction. A surgical operation lasting from fifteen to forty-five minutes is generally powerless to effect a lasting cure alone and demands for its ultimate success an after-treatment and care, quite out of proportion to the difficulties of the operation. The cutting of a small tendon for muscular wry-neck is a comparatively easy task and can be readily performed under the use of cocain; but the absolute cure of the wry-neck and all its disturbances and the prevention of its recurrence demand keen thought and patient toil and care for a long period. Mikulicz estimates that the average duration of the treatment of his cases of tuberculosis

*Read by Walter G. Stern, Cleveland Orthopedic Surgeon to Mt. Sinai Hospital before the Northeastern Ohio Medical Association, November, 1[illegible]02.

of bone and joints is over six months—three spent in the hospital and three spent at a sanitarium.

To treat a simple acquired deformity, Lane lays down the following rules:

(*a*) Improve general vigor and nutrition.

(*b*) Prevent the assuming of attitudes tending to increase the deformity.

(*c*) Adopt active exercise tending to correct deformity.

(*d*) Use mechanical or operative measures for the speedy relief of the deformity forces.

(*e*) Avoid use of instruments, braces, etc., which can at all interfere with the proper use or development of the parts.

It is not the purpose of this essay to advocate or describe special operations or procedures, but rather to hint, if possible, in general terms at the methods which are universally applicable and which underlie all conservative procedures in orthopedic surgery. My purpose is to encourage you in your insistance that all deformities, congenital or acquired, shall be under constant medical care and observation; to free you from the necessity of looking for aid from the surgical-instrument vender and to encourage you, and through you the parents, in the long wait so often necessary before the final results are seen. It is in this connection that hospital care, especially that obtained by institutions for the care of crippled children founded by the State is of incalculable benefit.

The operative procedures of orthopedic surgery are to-day on a level with those of general surgery. A rigid aseptic technic has done away with the necessity for the strictly subcutaneous incision and has made it possible to open up major joints without the fear of sepsis. Still the field of "bloodless" operations is not at all narrowed, if only for the reason that parents consent more readily to such procedures. Brisement force or modeling redressment instead of resection, osteoclasis in place of osteotomy, forcible manipulation instead of open incisions as in the reduction of congenital hip dislocation, are generally the operations of choice. Tendon transplantation has achieved such wonderful results of late years that in a recent paper (*New York Medical Journal,* September 5, 1903) I ventured the statement that at the present time there is no case of infantile paralysis, however old or neglected, which can not be improved by some of our more recent surgical measures. The forcible reduction of recent tubercular deformities, such as Cabot's operation for Pott's disease, has received a decided setback from the return of the deformity and from the death from general tuberculosis in a number of cases. The open operation for club-foot, such as Phelp's operation, is used only as a last resort, for the bloodless manual correction combined with tenotomies can generally relieve even the severer cases. However, as stated before, operative procedures in orthopedic surgery, no matter how keenly planned and skilfully carried out, are generally abject failures as far as permanent results are concerned, unless followed by after-treatment and care as keenly, persistently and dutifully performed as is the operation itself.

Mechanical measures are probably resorted to more than any others and demand for their complete success a knowledge of mechanics. An example of the difficulties in use of mechanical apparatus may be of interest. In contractures of the knee-joint, springs or rubber tension work best when the deformity is greatest, but they are wholly powerless in the last 10° of flexion or extension. Walking is not, however, perfect until extension is powerful at 4° to 5°. Consequently the ordinary braces and springs can not be used, and only on the scissors-brace, with the joints all made in the form of a large X with rubber or springs to get the bars together, is wholly successful. As most of our medical schools make no mention of this branch of mechanical therapeutics in their advertised courses, it is not to be wondered at that most physicians make use of the ready-made stock braces of the nearest instrument-house, or "send away to Chicago" where a twenty-five per cent. rebate awaits the fortunate physician who has a cripple patient. There still clings to the mechanical treatment the feeling that to manipulate apparatus, or to perform the necessary treatment, is in some obscure way outside of strict medical lines; and there are to-day in all sections of our country prominent members of our profession who still refuse to treat patients with deformities, but send them to some commercial instrument-maker.

The use of resistances and resistance exercises also come under the head of mechanical therapy. The hand of the mother or nurse makes an admirable resistance for a deformed child to exercise its limbs against, and attendants are for the most part easily taught. If this is not possible, or if more elaborate apparatus is necessary, the extremity can be fixed to a board and the required resistance obtained through straps, pulleys and weights. Here the child can be encouraged to perform prescribed exercises by attaching the clapper of a bell, a rattle or even an electric button to the strap, and the task converted into pleasant play.

Massage, baths, electricity, passive motion and gymnastic exercises are of the utmost benefit to prevent deformity; and at the same time one of the mainstays of all postoperative treatments. What can be accomplished by these measures alone can be readily seen from the report of a case by Leuf, in which the champion high jumper of the University of Pennsylvania (for that year) acquired his powers. In infancy he had a slight paralysis of the limbs and was treated for many years by systematic massage and exercise. Circulation in the paretic parts, inflammatory deposits about the joints, fibrous contractures and adhesions have all been noticed to improve when these measures alone have been persisted in. It was interesting to the writer to note in this connection that Lee had pamphlets on these topics translated into the vernacular tongues of India, where infantile paralysis seems to be unusually common.

Rest, fixation and unburdening are the *sina qua non* in the treatment of all acute diseases of the joints. Physiologic rest is the prime requisite in combatting pain and in the spontaneous cure of joint affections, but can hardly ever be obtained without mechanical aid, except in the spine where recumbency alone is able to fix the diseased part. In all cases rest can be obtained through fixation by means of simple extension, splints, sandbags, weights, and braces, or through the use of plastic materials which harden quickly, such as plaster of paris, celluloid, glass, glue, starch, clay, felt, wool or leather. My own preference has always been for the use of plaster of paris as the universal agent for fixation of bones or joints. When pure and freshly rolled upon crinolin bandages, it is easily applied, and can be made to fit snugly over any surface, no matter how irregular; it can be manipulated while setting, is cheap and durable, and fixes continuously in the right way all the time until the physician himself removes it. Braces, on the contrary, are expensive, must be made and applied by a second party and do not admit of accurate and continuous fixation because a third party can take them off at any time, and then may not get them on just in the right way. The case of F. B., age 6, suffering from Pott's disease is to the point. At the age of $3\frac{1}{2}$ years he suffered from Pott's disease and was treated by being placed on a hard bed. No deformity was visible nor did he have any abscesses or ulcers. After 14 months of recumbency he was placed in a plaster cast and allowed to walk about. The cast was renewed at intervals until six months ago when he was given a spinal brace. At the time the last cast was taken off there was no deformity nor abscesses. The mother says that she never could quite get the brace on right (nor do I blame her; there were 13 straps and buckles to fasten) and that in the last six months a large hump was slowly formed embracing the lower six dorsal vertebræ, and a psoas abscess can now be palpated on the right side; all are the result of imperfect fixation from the brace. Does plaster cause more atrophy than braces? Dr. Ely, of New York, whom I remember from Lorenz's clinic in Vienna to have been exceedingly antagonistic to the use of plaster, recently made a test in a case of double club-foot, encasing one side in plaster after manual correction and using a Taylor clubfoot brace on the other foot. At the end of a year the feet were both in good condition but the *one* with the *brace* was *no less atrophied* than the one encased in plaster.

Unburdening is a principle little discussed in America. It is only necessary in advanced cases, such as cervical Pott's disease or hip-joint disease in which simple fixation is not enough.

Pressure and counter-extension are used for the cure of deformity, as in the familiar treatment of the kyphos of Pott's disease. The treatment consisted of a roll placed under the deformity. Braces or a plaster cast are to be preferred to any form of counter-extension of pressure recumbency which dooms a child to lie in bed. Portable beds, frames or carts which can be wheeled out into the open air are, however, often useful when recumbency is absolutely necessary; in very young children a plaster shell moulded to the back answers the same purpose.

Education and gymnastics are of unmeasured value. To have conferred upon a previously functionless member new powers is not enough. The child must be taught to use them and keep up their strength. A flexor tendon transplanted upon the extensor side can be taught to act perfectly as an extensor, but if not kept in constant use will rapidly degenerate. In cases in which operative interference is not used education alone will often confer new powers. Dr. Seguin, of Paris, attempted to train the hand of an idiotic child. When he began the hand was small and wasted, the nails short and brittle and the muscles contracted; the fingers had no power or skill, and only automatic movements were possible. To make the hand follow a command was out of the question. The training began with shoulder exercises and when these were mastered were followed by purposive movements at the elbow, wrist and fingers. In 14 months' time the boy could drive nails, place pins in holes and perform all manner of difficult tests. In a case of inoperable *spina bifida,* which I saw last spring, the child had no control of her bowels, she could not make purposive movements with her feet and could only stand with the aid of braces and crutches. I placed her under the care of a dancing teacher who carefully taught her how to toe a mark, kick a ball and take dancing steps with the aid of her crutches. The teacher writes me that after six months the child can control the bowels long enough to get to her chair, can play hop-scotch accurately and dance a two-step with the aid of her crutches.

Shall we send our crippled children to hospitals and institutions? In Ohio the movement is just beginning, and from private funds, kindergarten schools and homes for crippled children are being founded. We still lack, however, the great essential, and that is the State institution with abundant means, in which such children can be cared for, brought up to useful manhood or womanhood, and at the same time be properly treated. There can be no question as to the great benefit to be derived from such homes, which are everywhere wonderfully successful. It is to be remembered, however, that orthopedic treatment is very expensive both to establish and to maintain ; splints, bandages, braces, apparatus, and the like, cost a great deal of money, and the children must be under constant treatment for a long time. Some of our local (Cleveland) institutions have fallen short of the very best results on this account alone, so that a well-equipped State hospital is *imperatively demanded.* A crippled child does not necessarily mean a helpless man, and society derives as much good or more from a home for crippled children than from an insane asylum, and the little ones ought to be wards of the State in their youth, rather than allowed to go uncared for and then forced to become inmates of our county poor-houses at the very age that the State homes for crippled children turn their charges out into the world as useful men and women. To have the long-continued care and after-treatment given by such a State home would often crown our operative procedures with success instead of failure. One example will suffice I am sure. David C., age five, suffered from infantile paralysis at the age of eighteen months. A club-foot developed and was operated on in Toronto, and David learned to walk ; but the case was neglected and the condition soon returned. One year ago I corrected the club-foot and transplanted several of the tendons and held the foot in the over-corrected position for a number of months by means of plaster casts. I then persuaded the mother to give the limb daily baths, massage, passive motion and exercises. The result was good, too good in fact, for the mother, a careless, ignorant woman, burdened with five other children, stopped the after-treatment as soon as she thought "Davie was oncet cured," and the limb soon wasted and relapsed into its former state. Both operations failed solely for the want of the proper after-care and attention. But where could David get these ? Our general hospitals turn out their patients as soon after the operation as possible, children's homes don't want cripples, visiting nurses do not give massage and a busy physician can not devote an hour each day to a single unremunerative case, who would present himself unkempt and dirty, provided the parents didn't find it too much trouble to bring him. Nor should the treatment of crippled children depend upon the ordinary exigencies of hospital or dispensary practice—as seen in the clinics of New York. Hip-disease, for instance, requires from one to three years of constant care and treatment, and yet how often is a child operated on or fitted with a hip-splint and dismissed to the home where it will lie neglected till a visiting nurse discovers it and sends the child to some other institution.

The prevention of deformity ought to be the aim of every well-minded physician, and can generally be accomplished, provided the physician and the patient have enough endurance and patience. Many acquired diseases, such as infantile paralysis, are not essentially deforming, and an early and correct diagnosis, with the proper management and care in carrying out the measures advocated in this essay, are sufficient to force the future growth of the child in the right direction.—*Cleveland Medical Journal.*

---

### A MEDICAL VIEW OF THE PREVENTION OF APPENDICITIS.*

Appendicitis, in the minds of a large and influential number of the medical profession, is a disease with which, as it progresses from the stage of catarrhal to septic inflammation, the surgeon alone is competent to deal. Undoubtedly, it must be granted by all physicians that surgery is a necessary and essential factor in the treatment of all cases of appendicitis which have progressed beyond the stage of non-septic inflammation. While during the last year some discussion has arisen among surgeons over the question whether or not to operate immediately upon making a diagnosis in all cases of appendicitis, and while to many the weight of the evidence seems to favor the Oschner plan of treatment, it is not within the province of this paper nor determination of this writer to bring this matter before the profession at this time.

Rather, I would call attention to a phase of the subject which reaches far into the etiologic and incipient stages of this grave disease, and to turn the eye of acute vision upon the processes of unseen disturbances which must seem to be harbingers of the pus and to inevitably, if allowed to proceed unarrested, carry the disease beyond the pale of internal medicine into the wonderfully useful field of operative surgery.

---

*Read by Alex G. Brown, Jr., M.D., Richmond, Va., before the Medical Society of Virginia during its thirty-fourth annual session, at Roanoke, Va., September 15-17, 1903.

Therefore it is not to the surgeon but to the internist or the family physician that this paper is addressed, for this class of medical men are the first to see appendicitis, in a large number of cases, in the initial stages and the premonitory states.

While it may appear a little inopportune to rewrite that already well-written subject, etiology of appendicitis, the matter of the primary cause of appendicitis is so germane to the question under consideration that I consider its presentation essential and so will review the predisposing and active causes of appendicitis briefly.

In the very embryological history of appendicular structure may be found one of the predisposing causes. For in the evolving stages of animal anatomy this functionless vestige, by inherited uselessness, has become a structure of low resisting power, having no vital organic function to perform, seems to be a ready victim of the weakest assailant. The position and the anatomical mechanism of the appendix also render that dependent structure a point of frequent attack. For the appendix has only one orifice, opening into a blind canal, curved, and more or less narrowed by traction of its mesentery, which is a part of the inferior layer of the mesentery of the colon. Traversing the free border of this fold of mesentery runs the single nutrient vessel of the appendix. Then, at the point of union of the large and small bowels, a pouch-like junction, lies the appendix, having a single narrow orifice, possessing a tortuous canal, and supplied by a single blood-vessel which courses its way along the free border of a suspensatory ligamentous band.

Keeping these facts in mind and granting the fact, now generally acknowledged, that a trauma, foreign bodies, typhoid fever, actinomycosis, tuberculosis, and malignant disease are but rarely the exciting causes of appendicitis, let us see to what various writers attribute this frequently occurring disease. After making a research of the literature at his command, Eagleson states that:

Hartly says, "Indigestion appears to be a cause, in that the matter in the intestines taken in excess, poorly digested, and badly tolerated, provokes a catarrh and anomalous constriction of the intestines."

Mynter says: "Constipation, diarrhea and digestive disturbances favor the development of appendicitis. These alone would not probably produce an appendicitis, but added to them an abnormal position of the appendix, by which stagnation is favored, and the result will be sooner or later an attack of appendicitis."

Morris speaks of gastric disturbances only in connection with the acute attacks of the disease.

Deaver says: "Of the exciting causes of appendicitis, from a clinical point of view, disturbances of digestion are the most important. Such is the pre-eminence of these in the etiology of appendicitis and with such constancy have been observed that it is unhesitatingly asserted that appropriate inquiry will elicit a history of such disturbances in nearly every case." He goes on to state that the indigestion causes an increased virulence of the intestinal bacteria which in turn cause the inflammation of the appendix. Fowler maintains the same theory and says: "In rare instances in which the development of the disease is preceded by digestive disturbances, the connection between the two occurrences may still be dependent upon bacterial agency."

Talmond says: "The erratic peristalsis produced by the presence of irritating material in the digestive tract determines the engagement of a stercoral calculus in the orifice of the appendix. This in turn gives rise to the symptoms constituting the first stage of the disease."

Anders says indiscretions in diet may precede a primary attack, and are of paramount etiologic importance in the recurrent forms of the malady.

Johnson says: "Appendicitis is probably often the final stage in intestinal indigestion which leads to catarrh of the cecum and consequent multiplication of bacteria with infection of the local peritoneum."

McNutt, in Loomis' System, states that indigestible and too highly seasoned foods, by provoking catarrhal conditions, by distending the bowels with fecal matter and gases, are also etiologic factors.

Osler refers to the frequency in which inflammation of the appendix follows the eating of indigestible articles of food.

Illoway maintains that this very grave affection is in a majority of instances provoked by constipation. This is brought about in three ways: First, by enabling fecal matter to pass into the appendix; second, by liquefying this and affording a culture-ground for bacterial propagation, with the resultant inflammatory processes; third, by leading to the formation of concretions, which become exciting causes of appendicular colic, ulceration, gangrene, or septic inflammation.

Vaudenbosshe recites a case which appears to show that dysentery, with its lesion in the cecum, by propagation of infection produces appendicitis. Patek says, very significantly, that many cases with the symptom-complex of gastro-intestinal irritation begin with an indifferent aim, and only secondarily become appendicular.

Vevey thinks the greater frequency of appendicitis may arise, aside from the more strenuous habits of the day, from a neglect of the old-fashioned habit of occasional purgation.

Robin sets forth the idea that appendicitis is a complication of gastro-intestinal derangement and he maintains that the first stage shows itself as gastric hyperesthesia with hyperchlorhydria secondarily, appearing in the form of acid fermentation throughout the gastro-intestinal tract, to be followed, thirdly, by stagnation in the cecum of abnormal, acid matters, which induce catarrhal processes, which, in turn, afford a good culture-ground for pyogenic infection.

From this array of witnesses may not the truth be established and the statement made, without fear of contradiction, that the causative factor of appendicitis is gastro-intestinal derangement? May not the forerunner and antecedent stage of appendicitis be an acute gastritis, with the diminished hydrochloric acid, excess of mucus, lactic, fatty acid, and a puddle of fermenting undigested food; or, may it not be a chronic gastritis, with deficient hydrochloric acid, undigested food-stuffs undergoing fermentation and decomposition, generating lactic, acetic, butyric acids and alcohol—a vicious ally to intestinal distress and disease?

Then, may not there follow duodenitis, and proceeding along the continuous intestinal coating of the small bowels, involving jejunum and ileum, may not a catarrhal inflammatory state be produced, creating, instead of a diarrhea (which nature with kindness usually brings on for relief of this condition and prevention of the evil results of appendicular envolvement), an accelerated peristalsis or, perchance, a more to be feared condition, antiperistalsis, which occurs later from colitis from the left transverse colon to ileocecal valve; and here, at this point of union of small and large bowel, the cecum, the storm-center, may not there be engendered a gaseous accumulation, creating an ileocecal colic, or an appendicular colic, whose great signal is pain?

This distinction of the caput coli with gases, fermentive matter, etc., fecal structure, all of which harbors and produces deadly germs of pus—produces congestion, tumefaction, catarrhal inflammation, septic inflammation, ulceration, or gangrene, which latter condition brings the appendix to that grave state where the surgeon alone can hopefully deal. Now the conclusion is plain—if the premises are true—that the prevention of appendicitis is possible, provided certain difficulties which render a present-day successful treatment of subject impracticable are overcome.

One great difficulty which would seem to embarrass the successful inauguration of a preventive treatment is the fact that the general practitioner, by some inherent law which seems to afflict the generalizer of any subject, fails to exert an acute perceptive diagnostic insight into the chain of disturbances which invariably, in large majority of cases, prevails in the gastro-intestinal tract and to read the true significance of the future possibilities for appendicular disease in that patient the victim of alimentary disturbances or cecal colic, or appendicular colic. Then, it would seem important to arouse the intellectual faculty to the realization of what it all means and to address remedial measures for the correction of disturbances therein. In this time, when gastro-intestinal disease appears to have reached the most excellent state of being almost an exact science, may it not be hoped that much can be done toward the annihilation of the causal factor of appendicitis to the surgical degree?

Another difficulty confronting this matter of prevention of appendicitis is that the patient upon whom our work is to be done is human and subject to the frailties of human nature and flesh—and he must eat and move and have his being—and even were the threatened appendicular envolvement prevented, may he not have a recurrence and this time prove so rapid as to be beyond all hope of prevention? To this grave phase of the matter, it might be urged that, if every internist or general practitioner were to constitute him, as it were, a specialist on gastro-intestinal diseases, and, through the magnified vision of the specialist, who sees so clearly the dangers of the abuse of the organs over which he has become the expert healer and guardian, and who can so emphatically and impressively state to the patient the grave and serious perils that threaten him and the proper treatment and habits to follow to prevent becoming the victim of these perils, then might we not hope to see the time when appendicitis would have a most formidable enemy in the intelligent co-operation of the patient over whom it has stood a pall of terror.

It is late in the diseased state when surgery is necessary—much has gone on before, to which the eyes of the doctor have been shut and realities to him unknown.

Every patient with gastric and intestinal complaints, acute or chronic, is a candidate for appendicitis, and that cæcum should be kept under constant surveillance. The patient should know the perils and dangers of a condition of this kind, and the physician should urge that the patient be treated until cured of that gastro-intestinal complaint—or else lay the responsibility of a future attack of appendicitis at the door of the unmindful and heedless patient.

My belief in the statements herein set forth has been made very great by a number of cases which have forced upon me these conclusions. In a very humble spirit I present this paper with the hope that the idea is not far from truth.—*Virginia Medical Semi-Monthly.*

---

### TYPHOID FEVER—THE MANAGEMENT OF THE CASE.*

All important in typhoid fever is a competent nurse. The room should be devoid of all unnecessary furnishing; bed linen and garments of occupants should be scrupulously clean and ven-

*Read by Samuel E. Earp, M. S., M. D., Indianapolis, Consultant in Practice of Medicine Indianapolis City Hospital, Protestant Deaconness Hospital, St. Vincent's Hospital, Indianapolis City Dispensary and Union State Hospital, before the Indianapolis Medical Society, December 15, 1903, being one topic of a Symposium on Typhoid Fever.

tilation should be the best. The bed should be one to insure the comfort of the patient and sufficiently wide to avoid accidents during delirium and yet be so constructed as to furnish due convenience to the nurse in the performance of her duties. A case record should be kept and in the absence of a trained nurse the physician should leave written directions.

At the onset the patient should be given a bath for the purpose of cleanliness and should be robed in a loose garment only. The bowels should be thoroughly evacuated; calomel or blue mass may be followed by some saline laxative to prevent absorption or reaction. This is perhaps the stereotype method and has but few opponents. However, castor oil I believe preferable, from the fact that it is more thorough in its action; it has all of the good therapeutic points and none of the bad ones. There is no chemical action takes place and it can be noted in the stools in about the same quantity in which it has been administered. Throughout the course of the disease, the bowels may be kept open by this agent. It may be made palatable by sandwiching it between sherry wine and glycerine, which I might denominate a castor-oil sandwich.

An enema of the normal salt solution may be used for the purpose of moving the bowels. Other benefits are derived from it during high temperature or when its therapy is indicated on account of other untoward symptoms.

If there be the slightest indication of tympanites, turpentine may be given in ten-drop doses each four hours; sometimes this is a valuable remedy throughout the disease and its hemostatic action may be demonstrated in case of intestinal hemorrhage.

The use of strychnia as a sustaining agent I consider more valuable than whiskey; it may be given throughout the disease, but the most important period generally is manifest during the later stages. I frequently use benzoate of guaiacol or the carbonate in five-grain doses, three times a day, although I am acquainted with the statement that an intestinal antiseptic in quantities to give results would injure the tissue with which it comes in contact.

Liquid food should be selected that will leave the least residue. It is not the amount taken that is important, but the quantity that is absorbed and assimilated. Milk may be used with barley water as a diluent. But if the stools show an undigested product, either less quantity should be taken or perhaps there should be a mixed diet. Buttermilk, koumiss, light broths and albumin water are valuable. The latter may be made by adding the white of three eggs to a pint of water and then straining the same. A patient as a rule will utilize about four ounces of food each three or four hours, but no food should enter the alimentary tract that can not be digested, and if for any reason the stomach is rebellious, we may resort to rectal alimentation. Water should be given freely and acts not only as a beverage but also as a therapeutic agent and aids very materially in sustaining the normal function of the kidneys; this I consider a very important feature. Mild cases need no other treatment than the foregoing, and some get well with much less care.

Perhaps it is unnecessary to specialize in reference to the conduct of the case in its later stages. Suffice it to say that the use of solid food should be resorted to very cautiously and the patient should not be permitted to sit up until after the temperature has been normal for about a week or ten days. There is one exception, however, to this rule, and that is the condition known as bed fever.

However, if the temperature approaches 103°, balneotherapy should be adopted in form of the cold bath, sponging or sprinkling; not because we are expected to reduce the temperature only, as some have claimed, but it absorbs body heat, relieves nervous symptoms, strengthens the heart, stimulates respiration, furthers elimination and is a method of cleanliness. The coal-tar products will, by no means, render as good service as the bath, even in the early stages. Furthermore, they are depressants, disturbers of nutrition, and do not act favorably upon untoward conditions of the nervous system. Better far would be the cool-air cradling suggested by Fenwick of London.

During the past year I have used guaiacol (Merck's) three drops each four hours internally, and locally 15 to 20 drops rubbed in the right iliac fossa each two to four hours. By this method I have seen a fall of temperature from 105° to 99° when all other methods had failed. Rigors are sometimes present, but apparently give no untoward results, and the heart is strengthened rather than depressed by this agent.

Dr. H. G. McCormick, of Williamsport, Pa., has used this method for a number of years and in the *Therapeutic Gazette*, August last, he reported 408 cases with a mortality of 5.4 per cent. He collected statistics from a number of hospitals in which 1,998 cases by other methods of treatments showed 265 deaths, a rate of 13 per cent. In case guaiacol does not give immediate results he gives 1-100 of a grain of nitroglycerine three times a day for two days.

I have been unable to confirm the abortive treatment used by the foregoing author in which much dependence is placed upon the germicidal action of acid sulphate of soda. It is claimed that it will prevent or ameliorate many of the untoward conditions and hence shorten the course of the disease. In reference to its use I might quote from the following letter, which I received from Dr. McCormick, December 3, 1903:

"The physiological action of guaiacol when applied locally has never been satisfactorily explained. There are several theories, yet none are entirely satisfactory. But that it does produce the results none who have given it any extended trial can or will deny.

"The acid sulph. of sodæ is the bi-sulph. sodæ (Merck) and none but this preparation should be used. It is given in 7 1-2 grain doses in an ounce of water every two hours and if you can commence this treatment during the first week of the attack you will render your case a mild and I believe a short one. Since writing the article you refer to I have used this remedy in about forty cases with the most gratifying results. If you or your medical friends have any doubt as to the power of this drug to destroy the bacillus of typhoid fever let them take water infected with the poison and add the sodæ in the strength I have given you and after thirty minutes see whether they can grow a culture from this water."

I have used acetozone in private cases and at the Indianapolis City Hospital. This new chemical was originally known as benzozone. I gave thirty grains dissolved in one-half gallon water to be used by the patient ad libitum, one and one-half quarts being consumed in twenty-four hours. I gave one to two grains each two hours in capsule form. It is claimed that as a germicide it is harmless and yet equals in efficiency bichloride of mercury in solution one part to 1000. The cases which made the best progress were mild in character, and we know that some cases get well with very mild treatment. In some cases it did not give me good results and at the present time I am not prepared to advocate this method.

Chantemesse has advocated the use of antityphoid serum. About five c. c. of sterilized cultures of typhoid bacilli grown in thymus bouillon are injected deep in the muscular tissue of the lateral gluteal region and for several days thereafter one c. c. He read a paper before the Egyptian Medical Congress in February, 1902, in which he stated that the statistics taken from the Paris Hospital showed a mortality of 19.3 per cent. In Dr. Chantemesse's Hospital where serum-therapy only was used there were seven deaths out of 186 patients, a mortality of 3.7 per cent. He claims that the injection should be given in 2 c. c. even if the diagnosis is uncertain. He asserts that the serum is anti-infectious and antitoxic and excites phagocytosis. However, some other statistics show a mortality of 4.7 per cent., 6 per cent. and 9.5 per cent., respectively. Special symptoms may be combated as they arise.

In vomiting, anesthesine in three grain doses, cocaine in one-half grain doses, salicylate of strontium in four grain doses, or iced champagne, each independent of the other.

In case the baths do not quiet the delirium, ice may be applied to the head, hot-water bottle, bromide of sodium, elixir of paraldehyde, chloralamid or hydrate of chloral. In case of hiccough and insomnia the remedies are familiar with every one.

I believe that methylene blue or urotropin are the most valuable agents in the cystitis of typhoid fever. Recently the latter remedy has been advocated as an enemy to the typhoid bacillus and has been suggested in the routine therapy.

Intestinal hemorrhage is quite frequently combated by opium, ergot and adrenalin chloride, but there is every reason to believe that it is wrong to lock up the bowels even in this condition. Of course every text-book advises opium, and this advice has been copied by compilers for many ages. After calling attention to this fact, McCormick says: "I admit that opium will control peristaltic motion of the bowel. But that there is such peristaltic motion to control after a hemorrhage in typhoid fever, I am not willing to admit. When a hemorrhage occurs of a sufficient amount to make an impression upon the strength of the body, it produces a general relaxation of the whole body, including the muscular system. This is well shown in the arterioles and capillaries, as a result of which we see a profuse perspiration. You would infer, however, from those who recommend the use of opium, that the muscular fibers of the bowel which cause the peristaltic movement were an exception to the other muscles of the body; and that instead of their force and strength being reduced by hemorrhage these were rather increased and stimulated, making it necessary to stop such action by some strong paralyzer of muscular action. And strange to say, this nonsense continues to be taught and written by those who are looked upon as leaders in modern and effective methods of curing disease. I do not wonder that their mortality is from 30 to 40 per cent. When secretions are locked up there may be decomposition of food, gaseous distention, pressure upon a weak ulcerated bowel, threatening perforation, and furthermore the pressure as a result of distention may cause paralysis followed by death."

At the Indianapolis City Hospital a method for which I do not claim originality gave good results. This method is the use of calcium chloride. It was used as follows: When the hemorrhage first appeared an enemata of one quart of water at 115° F. containing 60 grains of calcium chloride was used twice a day by the means of a fountain syringe which was held at an elevation of 20 inches. Thirty grains in solution is given per os twice a day. This treatment is kept up three to five days. At the beginning of the treatment some of the prepared liquid foods take place of the liquid diet and the use of the cold bath is abandoned. The diet should be simple and used sparingly for a few days. Occasionally a low vitality will require the use of nitrate of strychnia. Sometimes I use the ice-coil upon the abdomen, although it has some objectionable features. On the fifth day I stop the use of the calcium chloride and give five grains of salol or benzoate of guaiacol three times a day and also an emulsion of turpentine, fifteen drops to the teaspoonful, each three hours. Several ten-grain doses of subnitrate of bismuth may be used. In a few days the patient can again resort cautiously to the milk diet, if deemed advisable, and return to the use of the cold baths if there is a high temperature. Alcohol baths or cold packs may be used.—*Indiana Medical Journal.*

---

### TREATMENT OF ACUTE PELVIC INFLAMMATION.

BY PALMER FINDLEY, M. D., CHICAGO.

It is my purpose to confine my remarks to a discussion of the principles underlying the rational and conservative treatment of acute pelvic inflammation.

Gynecology will soon take its place alongside of obstetrics as an all but fixed science. Our knowledge of the pathology, diagnosis and surgical technique relating to diseases of women has reached such a degree of perfection that there can be no serious disagreement in our discussion of these sections of the subject.

I am impressed with the importance of a careful consideration of the principles involved in the treatment of acute pelvic inflammation, because of the great prevalence of the lesion in hospital and private practice, and because I see almost daily violations of the principles which I believe underlie all rational treatment. With no desire to appear extravagant in my statements, I am convinced that injudicious and untimely surgical intervention in the acute stage of pelvic inflammation is frequently responsible for the extension of the lesion, and for the death of the patient.

By early surgical intervention there is violated the long-established principle of giving rest to acutely inflamed parts. If our laboratory experience has taught us anything in connection with clinical work, it is that infections starting in the lower portion of the genital tract (vulva, vagina, cervix) tend to become self-limited if let alone, but as surely extend to adjacent and remote regions through excessive exercise, and through digital and instrumental interference. Bearing in mind that an infection confined to the cervix may be made to extend to the uterine body, thence to the tubes, and ovaries, and on to the peritoneal cavity by violating the principles of absolute rest, it behooves us to adopt with great caution any procedure that will disturb parts recently infected. To this end we are to prescribe the oft-repeated intra-uterine douche, particularly under high pressure; sterile salt solution injected under low pressure will dislodge blood-clots, and detached secundines, and by doing so it accomplishes all that is expected of an intra-uterine douche. Sterilization of the infected uterine cavity by means of antiseptic douches is no more possible than the sterilization of infected hands by the same sort of a solution allowed to gently flow over the hands for an equal length of time. There can be little virtue in the antiseptic douche beyond that of mechanical removal of non-adherent structures. This can be accomplished equally as well by the use of sterile salt solution and with less liability to injury from absorption of the fluid or from forcing it on through the fimbriated ends of the tubes into the peritoneal cavity. In no way is the principle of rest so frequently violated and with such disastrous results as by the indiscriminate use of the uterine curette. About eighteen months ago I discussed before this society the use of the curette in the treatment of acute puerperal infection, and I now repeat with added emphasis because of the experience of these intervening months. In the first place the uterine curette is useless in the treatment of the large majority of acute pelvic inflammations, and in the second place it is capable of untold harm.

*The curette is useless* at all times during the acute stage of pelvic infections save in those cases in which fetal structures are left in the uterus, and even in these cases the finger, placental forceps and Emmet's curette forceps are instruments of greater precision and of less danger. In all forms of acute pelvic inflammation other than puerperal, with retained secundines, there can be no place for the curette.

The *uterine curette is dangerous* because it disturbs infection which should be left quiescent and excites it to further extension and greater activity by the opening up of new avenues through which the infection may travel; by the breaking down of that protective zone which nature has built up in advance of the infection, and by the too frequent puncture of the infected uterus. These, in brief, are my reasons for condemning the curette in the treatment of acute pelvic inflammation.

The principle of rest is also violated by the practice of vaginal and bimanual examinations. Such examinations should be restricted to the minimum, because they not only cause pain, but serve a limited purpose and may do harm. Since surgical intervention is seldom required in the acute stage of pelvic inflammation, repeated examinations are rarely necessary. As a general rule it may be stated that where the general septic condition is not alarming and a careful pelvic examination does not reveal the presence of pus that can be drained through the vagina, it is well to proceed with the treatment along such general lines as rest, douches, hot or cold packs, careful regulation of the diet and bowels, and the relief of pain by anodynes, if necessary. After the acute symptoms have subsided, it is time enough to determine the exact extent of the lesion.

I have spoken somewhat at length of the principle of maintaining rest in the management of acute pelvic inflammation. I come now to the principle of free drainage. It is of the utmost importance to establish early and free drainage when a serious or purulent exudate is so located in the pelvis that it can be reached and drained through the vagina. I will venture the dogmatic statement that the abdomen is never to be opened for the purpose of drainage or for any operative procedure during the acute stage of pelvic inflammation.

Where vaginal drainage can not or for various reasons should not be established, we are constrained to wait the subsidence of the acute inflammatory reaction before resorting to an abdominal incision. It should be our purpose to delay the opening of the abdominal cavity until the infection has practically lost its virulence. Just when this is accomplished is not possible to say, as we well know from bacteriologic studies in connection with our clinical work. Gonorrheal pus tubes have been known to be sterile within six weeks of the initial infection, while on the other hand the gonococcus has been known to exist in the tissues for fifteen years. As a general rule it may be stated that surgical interference may be safely instituted in gonorrhea at an earlier period than in puerperal infections, because of the earlier sterilization of gonorrheal pus, and of the infrequency with which the gonococcus infects the general peritoneum. It is my practice to decline to open the abdominal cavity for the removal of infected appendages short of six months or a year from the time of infection. In the meantime much relief can be afforded, and sometimes a positive cure obtained, by following the general surgical principles in the treatment of acute inflammation in enjoining rest, freedom from all mechanical irritation, in establishing free drainage through the vagina, and in such palliative measures as hot douches, hot fomentations, glycerine and ichthyol tampons, catharsis, anodynes, and above all, normal saline solution under the skin, or per rectum, where there is general sepsis.—*Medical Herald.*

---

ERYSIPELAS, CAUSES AND TREATMENT.—MANLEY, T. H.

The early and effective treatment of any malady presupposes a proper understanding of its cause and the pathological changes in operation.

A considerable experience in the treatment of traumatisms of widely diverse characters, following various phases of infection, inflammatory changes, suppuration of gangrene, have long since convinced me, that though many writers still follow in the beaten tracks of established custom and label many of these as erysipelas, or local expressions of that malady, they bear no relation to it whatever, and that a broad distinction should be emphasized between erysipelas proper and those numerous local inflammations consecutive to wound infection, or arising *de novo*, as phlegmon of any type whatever, of furuncle, carbuncle or felon.

Erysipelas is a constitutional disease, an exanthem usually running through various definite stages much like variola, measles or scarlet fever, generally tending towards recovery, but like the above-named maladies may be accompanied or followed by various complications. It is always a fickle, treacherous affection. This malady rather belongs to the domain of internal medicine than surgery. It seems at times to be more or less contagious, but that a healthy wound is infected by one suffering from genuine erysipelas is extremely doubtful; no such instance has ever come under my observation; now, after nearly thirty years in medicine, and having seen several hundred cases of the disease.

*Causation.*—The exact etiology of erysipelas yet remains very obscure; season, sex, age and systematic conditions all play a role. Fehleisen, in 1881, believed he had isolated the specific germ of the disease. But in spite of extensive bacteriological investigation on this subject, there is no unanimity or common accord of opinion on it yet; on the contrary very much confusion and contradiction are still obvious in the very latest works of our most eminent investigators.

In former times the disease was regarded as depending on defective hepatic action, or intestinal fermentation, impoverishment of, or impurities in the blood, disturbance in the nervous system, inheritance, etc. Now in order to support the germ-theory the causes must be proven as all extraneous, which can only operate through open wounds. This view is utterly untenable unless we confess to constantly and unconsciously traumatising our bodies in order to provide atria for the specific micro-organism. But on this phase of the subject, more further on.

What do we understand by erysipelas?

The ancients confounded erythema, eczema and a host of other cutaneous affections under this head, but for the past century or more its essential features have only been embraced in the definition of the term. Nevertheless there is by no means yet a common consensus of opinion as regards its etiology or pathology, it was thought well in this connection to briefly quote the definition of the malady as set forth by a few of our best known authorities, to wit, as follows:

Larry : Erysipelas depends on predisposition and local influences.

Campbell De Morgan : There is no doubt now but erysipelas begins in the blood and manifests itself in altered local conditions with a general febrile reaction.

Abernethy : It is the state of the constitution which determines the character of the local disease. I'll be hanged if erysipelas is not always the result of a local and disordered state of the digestive organs.

Fergusson: Erysipelas is a treacherous, uncertain malady. It bears a close relation to the ordinary type of severe inflammation.

Billroth: I freely admit my inability to prove that erysipelas is a specific poison. It may develop in the body from substances which have no relation to extrinsic influences.

Volkman: A local disturbance dependent on the influence of a special poison.

Heckteon: Erysipelas is due to a wound implication, caused by the inoculation of the specific streptococcus erysipelas.

Senn: Erysipelas is always traumatic and dependent on the specific streptococcus.

Nothnagel: The disease is due to the introduction of the well-known organism into the skin, or mucous membrane.

Grunewald: Erysipelas begins preferably in some insignificant abrasion of the surface and is probably propagated more by fession fungi already in the body than by freshly imported germs.

Some of us who have seen considerable of this disease were impressed with the belief that pathological conditions of the system were a factor in the etiology of this malady. Let us see how far we are in accord with our more modern authorities on this point; and how far the doctrine Fehleisen is supported; that only one specific coccus, introduced through a wound, can produce the disease.

Delafield and Prudden deny any specific properties to Fehleisen's microbe, and say: "The most common excitant cause of erysipelas is the streptococcus pyogeneus; this was thought by Fehleisen to have a definite relationship to the disease, but it is now proven identical with the ordinary streptococcus." (Handbook of Pathology, p. 185.)

In the *Courier of Medicine* for September, 1902, we find the following: "Marmorek claims that all pathogenic streptococci are identical. He bases his claim on three common characteristics that he has found present in all the bacteria taken from forty-two diseases. These peculiarities were: The production in vivo of hemolysis in rabbit's blood, the inability to grow upon the filtrate of their own cultures, and the immunization of animals by Marmorek's anti-streptococcic serum. The immense amount of labor in trying to separate different species of streptococci is, therefore, wasted, and clinicians will be compelled to find some other excuse why the anti-streptococcic serum does not cure."

Fehleisen: Erysipelas is now indisputably proven to depend upon a scientific micro-organism, the streptococcus erysipelatis.

Crocker: Erysipelas may be produced by either the streptococcus or the staphylococcus; pus organisms are the *fons et origo mali*.

Warren: It is a specific disease dependent on trauma and a specific streptococcus.

Hajek: Erysipelas may be produced by any of the pyogenic organisms.—*The Medical Examiner and Practitioner.*

---

### LABORATORY FOR STUDY OF ABNORMAL CLASSES.

There is an effort in our country to establish at Washington a laboratory to study the abnormal classes. This effort has been especially endorsed by the medical profession of our country. This is in reality an endeavor to develop a work already begun in the Bureau of Education at Washington. In a little corner of this bureau this work has been carried on under many difficulties for the last ten years; it has produced six government publications on crime and related subjects, which have become almost as well known in Europe as in this country. Some of these works are used as reference or text-books in our universities, and some have been translated into different languages. In brief, this work has been well received by the medical and scientific world, notwithstanding it is somewhat of a pioneer nature, and, therefore, is liable to encounter special difficulties in the way of prejudice, ignorance, misinterpretations, in addition to the unavoidable mistakes to which all pioneer work is subject.

In the last Congress a bill to establish a laboratory to develop this work was unanimously reported by the judiciary committees of the House and Senate, committees whose members are some of the leading jurists and statesmen of this country. In addition to such endorsements, this work in the Bureau of Education has been considered carefully by numerous religious, legal and scientific associations of highest rank, and resolutions have been passed by them favoring its development. The International Congress of Criminology of Europe, after discussing thoroughly the plan of this work, passed a resolution commending such work, and sent copies of their resolution to leading members of Congress and of the executive branch of the government. As this Congress consists of the chief specialists of the world in these lines, this alone should be sufficient endorsement. We have mentioned somewhat in detail the support this work has, to show plainly a most peculiar if not astonishing condition of things. Instead of this work being encouraged by the Commissioner of Education, it has not only been discouraged and quietly opposed, but even dropped out of the government on his recommendation. In doing this he practically sets himself up not only against the medical profession of this country, but the highest authorities in these lines in the world.

When it was asked on the floor of the House why the appropriation for this work was omitted, the chairman of the committee in charge of the matter said that his committee did not go into the merits of the matter at all, but simply accepted the recommendation of the Commissioner of Education. This action of the committee was, of course, the usual one, since the recommendation of any head of a bureau is followed, especially when reductions are asked for.

Here, then, we have a federal officer in the name of the government setting himself against the opinion and the desires of large bodies of learned associations all over our country. It is well known that the present Commissioner of Education has practically no scientific training, his life being devoted mainly to metaphysics and to educational matter. Whatever knowledge of criminology, medicine or science he may possess is theoretical or book knowledge. This is not the kind of knowledge that gives weight to opinion. And yet the opinion of this official with second-hand knowledge is allowed to defeat the wishes of numerous learned associations and specialists who have practical acquaintance with the field of work. It must be remembered also that these men are thoroughly educated men, many of them much better than the federal chief himself.

In dropping a scientific position out of the Government in this way, the commissioner did an unprecedented act, and scientific men in the universities of our country regard it as a most effectual way of telling the public that such work, no matter how much desired and highly recommended, is not wanted in the government.—*The Interstate Medical Journal, St. Louis, Mo.*

---

### THE MOUTH AND ITS CARE IN THE SYPHILITIC.*

When entering upon the management of every case of syphilis, the mouth is to be considered of first—if not greatest—importance. Mucous patches are of more frequent occurrence in persons who use tobacco in any form, but cigarettes appear to me to be the most irritating of all. It is needless to state that alcohol has an ugly influence, not only over the local conditions, but the general as well. I have found that after all local irritant influences have been removed, that under constitutional treatment this manifestation of the disease as a rule speedily subsides. No matter at what stage of the disease, iodides remove mucous patches more rapidly than any other drug with which I am familiar, but it requires mercury to keep the condition in abeyance. Too frequent application of the usual remedies used for this condition is to be condemned. I have found hot water of greater value than all the drugs combined when used locally for the relief of this condition; hence, I would recommend its use freely many times daily. Mercury, being regarded as a well-nigh indispensable article in the management of this class of cases, and producing as it does ptyalism in an exaggerated and sometimes horrible degree, it behooves every practitioner to give particular attention to this accident; the comfort and general welfare of the patient demand this, and no physician does his full duty unless due cognizance of this fact is taken into consideration. I believe with Clevenger, that aside from the chemic effect of mercury that its chief effect is mechanical and that ptyalism is invariably produced by an accumulation of metallic

*Read by C. Travis Drennen, M.D., Hot Springs, Ark., before the Mississippi Valley Medical Association at Memphis, Tenn., October 6, 1903.

mercury in the lesser blood-vessels supplying the jaw. We are usually told by the various writers with whose teachings I am familiar, that the patient should consult a competent dentist and have the mouth put in proper condition, and thereafter that the patient should use the tooth-brush and some antiseptic mouth-wash, in order to keep the mouth in proper repair. Such advice is well and good, but does not go far enough. If good results are to be obtained, attention to detail must be carried out in all these cases. We all know how hard it is to get the average patient to do even the simplest things of life, and he can not see the necessity of the foregoing procedure unless the physician makes it a point and insists that this one thing, of all things, must be assiduously carried out. It is not enough to tell our patients to care for the mouth after the fashion in which they are usually instructed; but, as before stated, we should go into detail. I make it a rule, and have for many years, to send every syphilitic, if the mouth be not in perfect condition, to a competent dentist, and upon his return I tell him that before and after every meal, it is absolutely necessary to take a tooth-brush and brush the teeth thoroughly. If bleeding should take place so much the better—it removes engorgement—and if little globules of metallic mercury are plugging some of the little blood-vessels, in this way they will be removed. If the gums are tender, spongy, and have a tendency to bleed easily, in addition to the above instructions, personally, I make it a rule to take alcohol and apply it once daily thoroughly to the tender and swollen gums. Now, it sometimes becomes necessary, in order to carry this method out as it should be, to first make local applications of cocaine prior to applying alcohol; but no matter what is necessary, this is invariably done. Again, the patient may carry out faithfully all of the foregoing, and yet ptyalism results, if one other procedure be left undone, i. e., when finished with the brushing of the teeth, if the patient will take the finger or thumb and carry out a systematic massage of the gums for several minutes, not less than half a dozen times daily, or more often, we need have no fear of ptyalism.

I wish to say in conclusion, that during the past seven years this has been my custom, and during that time I have used ungt. hydrarg., in doses ranges from a half dram to two-thirds of an ounce daily, externally, sometimes for weeks at a time, and not a single well-developed case of ptyalism has come under my observation as a result of the foregoing treatment.—*Medical and Surgical Monitor.*

